# Identification of Candidate Chromosome Region Related to Melon (*Cucumis melo* L.) Fruit Surface Groove Trait Through Biparental Genetic Mapping and Genome-Wide Association Study

**DOI:** 10.3389/fpls.2022.828287

**Published:** 2022-04-05

**Authors:** Xin Du, Hongyu Liu, Zicheng Zhu, Shusen Liu, Zhengfeng Song, Lianqin Xia, Jingchao Zhao, Feishi Luan, Shi Liu

**Affiliations:** ^1^Key Laboratory of Biology and Genetic Improvement of Horticulture Crops (Northeast Region), Ministry of Agriculture and Rural Affairs, Northeast Agricultural University, Harbin, China; ^2^Horticulture and Landscape Architecture College, Northeast Agricultural University, Harbin, China; ^3^Shouguang Sanmu Seeding Co., Ltd., Shandong, China; ^4^Qinggang Ruixue Agriculture Co., Ltd., Heilongjiang, China

**Keywords:** melon, fruit surface groove, BSA-seq, molecular marker, GWAS

## Abstract

The melon fruit surface groove (fsg) not only affects peel structure and causes stress-induced fruit cracking but also fits consumers’ requirements in different regions. In this study, genetic inheritance analysis of three F_2_ populations derived from six parental lines revealed that the fsg trait is controlled by a simple recessive inherited gene. Through bulked segregant analysis sequencing (BSA-seq), the *Cmfsg* locus was detected in an 8.96 Mb interval on chromosome 11 and then initially mapped to a region of approximately 1.15 Mb. Further fine mapping with a large F_2_ population including 1,200 plants narrowed this region to 207 kb containing 11 genes. A genome-wide association study (GWAS) with 187 melon accessions also produced the same chromosome region for the *Cmfsg* locus. Due to the rare molecular markers and lack of mutations in the coding and promoter regions of the 11 candidate genes in the fine-mapped interval, we conducted *in silico* BSA to explore the natural melon panel to predict candidate genes for the *Cmfsg* locus. A 1.07 kb segment upstream of *MELO3C019694.2* (annotated as the AGAMOUS MADS-box transcription factor) exhibited a correlation with the grooved and non-grooved accessions among the F_2_ individuals, and a natural panel consisted of 17 melon accessions. The expression level of *MELO3C019694.2* in the pericarp was higher in grooved lines than in non-grooved lines and was specifically expressed in fruit compared with other tissues (female flower, male flower, root, and leaf). This work provides fundamental information for further research on melon fsg trait formation and molecular markers for melon breeding.

## Introduction

Melon (*Cucumis melo* L.) is a worldwide economic cucurbit crop with multiple nutrients and delicious flavor. Uniform size and shape, high yield, and good quality are necessary conditions for elite varieties (Zalapa et al., 2007). Consumers have a high degree of melon acceptance, which is mainly affected by external characteristics (Pitrat, 2016). In recent years, a large number of genetic studies have focused on the external characteristics of melon, including peel color and fruit shape (Feder et al., 2015; Pereira et al., 2018; Oren et al., 2019). Diaz et al. (2011) and Monforte et al. (2014) reviewed the literature on QTL mapping in melon and inferred 9 meta-QTLs (five for fruit size and four for fruit weight) that could be detected in multiple melon mapping populations. Pan et al. (2020) sorted out the QTL analysis of fruit size, shape, and fruit weight of cucumber, melon, and watermelon, obtained approximately 150 consistent QTLs, and found that the homology of melon fruit shape gene is widely conservative in structure and function.

The melon fruit surface groove (fsg) is an important fruit morphological feature for consumer selection, which is mainly reflected in the fruits of some cucurbitaceae varieties and can be described as a suture from the flower end to the stem end (Zhao et al., 2019; Paris, 2000). The characteristics of fruit surface grooves affect the peel structure and fruit cracking of melon. Melon fsg can accelerate the browning of melon peel, and peel browning is the main factor leading to post harvest loss. Park and colleagues observed the area of melon fruit surface groove histologically and found that the volume and tightness of melon epidermal cells had changed during cold storage. The groove area on the fruit surface destroys the cuticle, reduces the protection of epidermal wax, and accelerates the browning of the pericarp (Park et al., 2020). The fruit surface groove also has a significant negative correlation with fruit cracking, and melon with fsg does not easily crack. The integrity of mature melon fruit without cracking is conducive to the transportation and storage of melon and increases the potential market value (Wang et al., 2018). Melon fruit groove width was negatively correlated with the edible part (Yousif et al., 2011). Since the reference genome of melon was published in 2012 (Garcia-Mas et al., 2012), some scholars have studied the genetics of melon fruit surface grooves. Zhao et al. (2019) predicted *MELO3C019694* as a melon fruit suture candidate gene by a GWAS strategy with a large number of melon germplasm resequencing data. Hu et al. (2019) also used a similar method to study the external characteristics of melon. Three SNP loci significantly related to the depth of the fruit surface groove were found on chromosomes 5, 7, and 11. Harel-Beja et al. (2010) identified a total of 44 fruit QTLs by constructing a genetic map, and among the three loci related to the melon fsg trait that were screened, two were located in linkage group 6, and one was in linkage group 11 and found that two loci were also colocalized with stripe traits. Ramamurthy and Waters (2015) studied the rib dept. of snake melon (*Cucumis melo* sub. *melo flexuosus*) and identified a QTL related to it at 0–16 cM on chromosome 11. Recently, Kishor et al. (2021) used 48 melon varieties to detect genome-wide SNPs by genotyping-by-sequencing (GBS) and then combined them with GWAS analysis, 18 important SNPs related to various morphological features were detected, and two SNPs related to fruit groove width were found on chromosomes 1 and 8. Based on the above research, we found that the melon fsg trait was an important trait in both melon fruit development and consumer choice. To date, some publications have shown that the candidate gene of fsg is located on chromosome 11, but fine mapping and candidate gene analysis are still lacking.

Previous studies have found that MADS-box family members have different regulatory functions in plant flowering time control, flower meristem maintenance, flower organ determination, wheel crack zone formation, fruit maturation, embryonic development, and vegetative organ development (Dreni and Zhang, 2016; Nakatsuka et al., 2016; Li et al., 2017). MADS boxes are expressed in the ovule, developing carpel, and developing fruit peel (Busi et al., 2003; Hileman et al., 2006). Floral organ development refers to the process of plant flower primordia developing into mature flower organs (Pelaz et al., 2000). In recent years, many studies have shown that the C-type functional gene AGAMOUS (AG) among the members of the MADS-box gene family plays an important role in the development of floral organs (Wei et al., 2020). The fruit of *A. thaliana* is a capsule with longitudinal cracking and persistent medial compound leaves, which are called siliques. The histogenesis and fragmentation of *A. thaliana* fruit are related to the action of specific transcription factors. In *A. thaliana*, several MADS-box transcription factors are necessary for different aspects of fruit development; these transcription factors include SHATTERPROOF1 (SHP1) and SHP2, which act redundantly to specify valve margin identity and the dehiscence zones in the fruit (Liljegren et al., 2000). The expression of SHP 1/2 in *Arabidopsis* is strictly located in the valve marginal region by inhibiting the expression of FUL in adjacent valve regions and inhibiting the expression of REPLUMLESS (RPL) in adjacent valve regions. SHP promotes the expression of two bHLH factors, INDEHISCENT (IND) and ALCATRAZ (ALC), which guide the production of the silique lignified layer and separation layer (Rajani and Sundaresan, 2001; Liljegren et al., 2004). Tomato AGAMOUS-LIKE 1 (TAGL1) is a MADS-box transcription factor gene belonging to the PLENA (PLE) lineage within the AGAMOUS (AG) branch. TAGL1 and FUL1/FUL2 are homologs of *Arabidopsis* SHATTERPROOF (SHP) and FUL, respectively, and their inhibition resulted in a mature defect phenotype that was partially similar to the *rin* mutant fruit (Shima et al., 2013). Mutation complementation and antisense gene expression analyses have demonstrated the function of MADS-RIN in regulating fruit ripening (Vrebalov et al., 2002). The MADS box has also been identified in several other fruits and is involved in early fruit development. A bilberry (*Vaccinium myrtillus*) FUL homolog, Vm-TDR4 (Jaakola et al., 2010), and a strawberry SHP homolog, Fa-SHP (Daminato et al., 2013), are associated with fruit development.

## Materials and Methods

### Plant Materials

**Figure 1:**
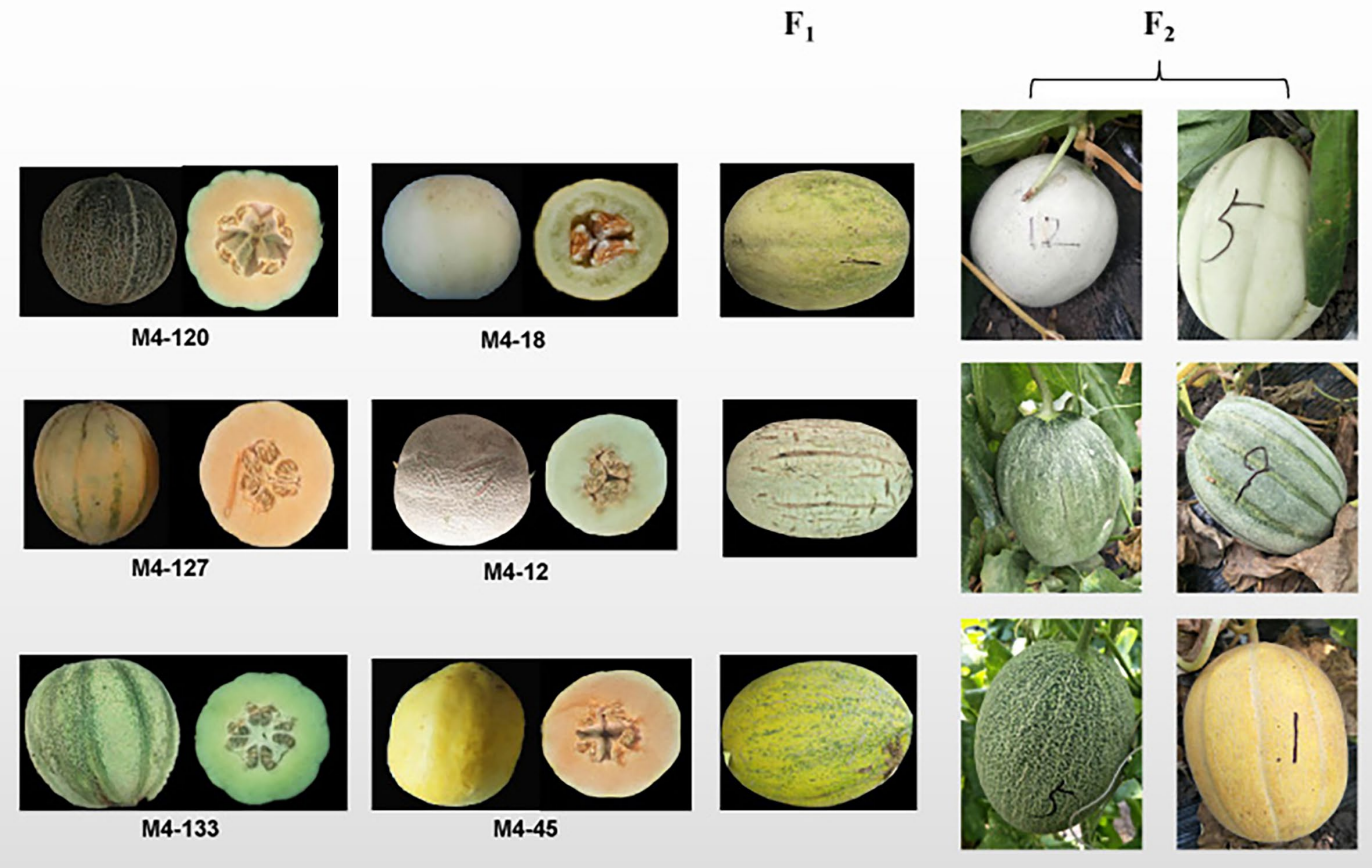
Parental lines of the three genetic populations and the main phenotypes of the F_1_ and F_2_ populations.

Three F_2_ segregate populations were derived from three grooved ssp. *melo* inbred lines (M4-127, M4-120, and M4-133; Figure 1) and three non-grooved inbred lines (M4-12, M4-18, and M4-45; Figure 1) containing 187, 183, and 160 individuals, respectively. All the experimental materials were grown in a greenhouse at the Xiangyang Experimental Agricultural Farm of Northeast Agricultural University, Harbin, China in the spring of 2020. The three F_2_ populations were used for genetic inheritance analysis in 2020. The F_2_ populations derived from M4-12 and M4-127 were used for BSA-seq, initial mapping and fine mapping. In the spring of 2021, a total of 1,200 F_2_ individuals derived from M4-127 and M4-12 were planted for recombinant selection. The selected recombinants were self-pollinated to obtain F_2:3_ families for recombinant F_2_ genotype identification.

### Evaluation of fsg Trait

During the fruit maturity period (30 ~ 45 days after pollination, DAP), the fsg trait was investigated and photographed. The fsg trait data were investigated by direct observation. Due to the deep and shallow differences in the phenotype of fsg in F_2_ fruit, cross-section should be observed along the fruit center axis to determine whether the fruit has fsg. The investigation traits were divided into two kinds of groove and non-groove (Supplementary Figure S1).

### BSA-Seq and Initial Mapping

The young, disease-free leaves of the M4-12 and M4-127, F_1_, and F_2_ populations were collected 15 days after seeding for DNA extraction with the modified CTAB (cetyltrimethyl ammonium bromide) method (Murray and Thompson, 1980). All F_2_ individuals from the M4-12 × M4-127-F_2_ population were numbered. A total of two bulked samples were arranged by mixing an equal proportion of DNA extracted from 20 grooved and 20 non-grooved F_2_ plants. The two gene pools and the genomic DNA of the parental lines were resequenced at the BGI Research Institute using the Illumina HiSeqXten platform (with at least 20× coverage genome sequencing depth for each sample) for BSA-seq analysis. The resequencing data analysis process was referenced by Takagi et al., 2013. Raw data obtained by sequencing were subjected to quality control to obtain clean data. The clean data were compared to the melon reference genome (DHL92 v3.6.1, http://cucurbitgenomics.org/ftp/genome/melon/v3.6.1/) through Burrows Wheeler Aligner (BWA) software (Li and Durbin, 2009). Different homozygous sites in the two gene pools were extracted to calculate the single-nucleotide polymorphism (SNP) or insertion–deletion (InDel) variation frequency and analyze the chromosome segment significantly associated with the fsg trait. To intuitively reflect the region of the difference between the two offspring pools, the difference between the two offspring SNP indices was calculated. One Mb was selected as the window with a 1 kb step size, and the average value of Δ (SNP index) in each window was calculated to reflect the distribution of Δ (SNP index). Combined with population type and offspring pool number, 1,000 permutation tests were performed, and 95 and 99% confidence levels were selected as threshold lines. The detected area above the threshold is designated as the chromosome segment primarily related to the fsg trait.

Subsequently, 500 bp before and after the InDel (Insertion and Deletion)/SNP-site sequences were extracted from the resequencing data for molecular marker exploitation. At least 6 bp InDel variation sites were selected for primer design. PCR products were separated by 8% denaturing polyacrylamide gel electrophoresis and silver staining for genotype data collection. SNPs in the restriction enzyme cutting sites were converted into CAPS (cleaved amplified polymorphic sequence) markers through SNP2CAPS software with 10 restriction enzymes (*Hin*dIII, *Bcl*I, *Xho*I, *Nde*I, *Eco*RI, *Eco*RV, *Alu*I, *Taq*I, *Xho*I, and *Dra*I). The PCR products and enzyme-digested products were detected by 1% agarose gel electrophoresis. Primers exhibiting codominant polymorphisms among the parental lines and the F_1_ generation were selected for genotyping. InDel and CAPS markers were designed with Primer Premier v6.24 software based on the resequencing data from M4-12 and M4-127. SNPs that could not be converted into CAPS markers were designated Kompetitive Allele-Specific PCR (KASP) markers and genotyped at the Vegetable Research Center of the Beijing Academy of Agricultural and Forestry Sciences. Individuals with recessive trait in the F_2_ population (M4-12 × M4-127-F_2_) planted in 2020 were selected and genotyped to detect the recombination events for initial mapping.

### Fine Mapping of the fsg Trait

A total of 1,200 F_2_ individuals derived from M4-127 and M4-12 were sown in the greenhouse in the spring of 2021. Each individual in the F_2_ population was numbered, and young leaves were collected 15 days after seeding for DNA extraction. The two flanking markers of the initial mapping region were used for recombinant selection. All recombinant plants were transplanted to the greenhouse of the Xiangyang Experimental Agricultural Station of Harbin Northeast Agricultural University and self-pollinated to obtain F_2:3_ families. The fsg trait was investigated when the fruits were mature. F_2:3_ families (at least 20 plants for each family) from the recombinant individuals with domain traits were planted in the autumn of 2021 to distinguish the genotypes of homozygotes and heterozygotes. New markers were also developed to detect recombination events in the initial mapping section to narrow down the initial mapping area.

### Genome-Wide Association Study of the fsg Trait in Melon

A total of 187 out of 297 melon lines and their resequencing data from our previous publication (Liu et al., 2020) were selected for the GWAS analysis. The phenotype of the fsg trait was assessed from photographs of mature fruits taken in 2015 and 2016. A total of 2,045,412 high-quality SNPs (MAF > 0.05) were used for associated signal detection with a compressed mixed linear model (MLM). *p* values of association for each SNP and an individual trait were calculated with TASSEL 5.0.^1^ The reference genome used for GWAS analysis was DHL 92 v3.5.1.^2^ The Manhattan and Q-Q plots were graphed with TASSEL v5.0 and qqman v0.1.8^3^ through R package 3.6.1.

### Candidate Gene Prediction With *in silico* BSA and Expression Analysis

Candidate gene function annotation was performed according to a reference melon genome (DHL 92 v3.6.1). Nucleotide mutations and structural variations (in both the coding and promoter regions) in the candidate genes were first compared between M4-127 and M4-12 based on the resequencing data with Integrated Genomic Viewer (IGV) software.^4^ We also selected genome resequencing data from other melon accessions (2 with the grooved: M4-120; M1-96 and 3 non-grooved: M4-12, M4-18, and M1-7) to compare genome sequences in the fine-mapping region for the variation polymorphisms between grooved and non-grooved melon accessions.

Ovaries and pericarp tissues with and without grooves (at the fruit mature stage) were collected from M4-127 and M4-12 for total RNA extraction and gene expression pattern analysis. RNA extraction was performed using a plant total RNA isolation kit (Sangon Biotech) according to the manufacturer’s instructions. SYBR Green Master Mix was used as the fluorescent reagent. *MELO3C008032.2* was used as the actin gene. Primers for the actin gene and candidate gene are listed in Table 1. Specific transcript amplification was verified by the presence of a single peak in the melting curve obtained after the amplification reaction was complete. The expression levels of the potential genes were analyzed with the 2^−ΔΔCt^ method (Livak and Schmittgen, 2001).

**Table 1:** Primers in this research.

Control gene primer	*MELO3C008032.2*	F:5ʹ-GTGACAATGGAACTGGAATGG-3ʹ R:5ʹ-AGACGGAGGATAGCGTGAGG-3ʹ
qRT-PCR primer	*MELO3C019694.2*	F:5ʹ-GGGAAGTGGAACTTCAGAGC-3ʹ R:5ʹ-TCGTTTGCTGTTGTTGTTGC-3ʹ
MAS PCR primer	PCR	F:5ʹ-CATGCCTGCAAGTAAAGACA-3ʹR:5ʹ-ATGCGGAGGGTACAATAAGA-3ʹ

### Molecular Marker-Assisted Selection (MAS) for the *Cmfsg* Locus

A pair of primers (Table 1) was designed based on the sequences flanking the 1.07 kb deletion region (chr11: 24,149,100-24,150,171). This chromosome segment was amplified from 15 melon lines (9 with grooved traits and 6 non-grooved lines) and detected by 1% agarose gel electrophoresis to check the correlation between phenotype and genotype.

### Statistical Analyses

A statistical field survey of the fsg trait data was carried out with Microsoft® Excel 2007. Genetic analyses and evaluations of differences in gene expression were performed using SPSS v.21.0 software (SPSS Inc., Chicago, IL, United States). Prism 7.0 software was used (GraphPad Inc., La Jolla, CA, United States) to prepare figures.

## Results

### The fsg Trait in Melon Is Regulated by a Single Recessive Gene, BSA-Seq, and Recombinant Events Delimiting the *Cmfsg* Locus Into a 207-kb Region

**Figure 2:**
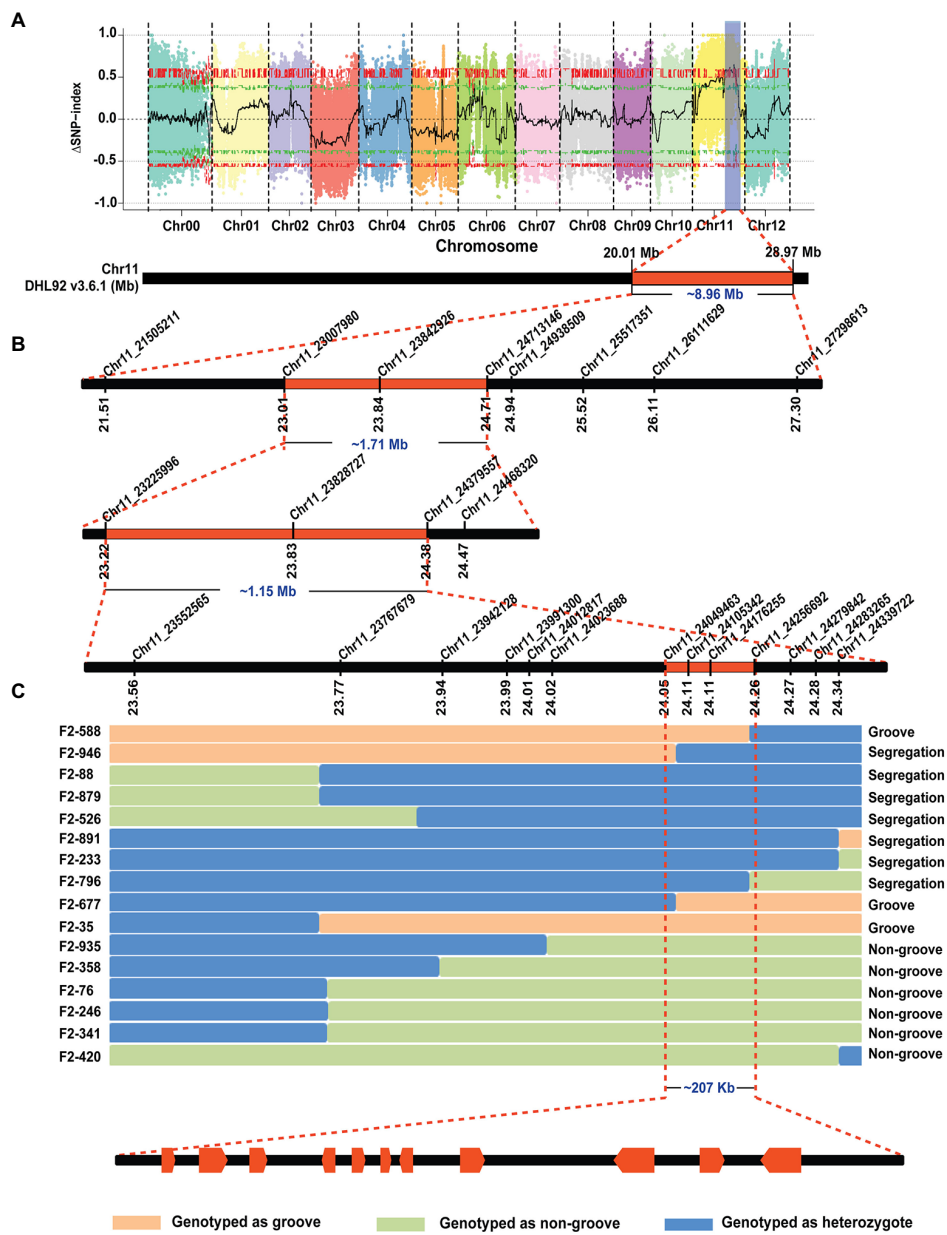
BSA-seq analysis and fine mapping of the melon fsg trait. **(A)** BSA-seq results, the chromosome region related to the fsg trait, **(B)** initial mapping of the fsg trait. **(C)** Fine mapping of the fsg trait.

The F_1_ generations from three hybridized combinations exhibited a smooth pericarp without fsg. In the three F_2_ populations, the ratios of fsg to nonfsg populations were 51:136, 40:143, and 36:124, which was consistent with the 1:3 ratio (Figure 1; Table 2) indicating that the fsg trait in melon is controlled by a recessive gene. Genome resequencing produced a total of 22.738 Gb of raw data. After filtering low-quality and short reads, M4-12 produced 11.385 Gbp of clean data, and M4-127 produced 11.352 Gbp of clean data (Q30 ≥ 88.28%). The clean data were compared to the reference genome through BWA software with comparison rates of 94.88 and 97.24%. A total of 75,902,530 and 75,682,054 clean read pairs were generated from the non-grooved pool (24.54× depth coverage) and grooved pool (24.77× depth coverage), respectively. Finally, 2,588,595 SNPs and 544,356 InDels were used for BSA-seq analysis. The ΔSNP index was calculated and plotted against genome position. A ΔSNP-index signal (with a ΔSNP-index value >0.5) related to fsg was detected on chromosome 11 spanning approximately 8.96 Mb (from 20013001 to 28966000 bp; Figure 2A).

**Table 2:** Genetic analysis of the fsg trait among F_2_ segregated populations.

Genetic population	Total	Groove	Nongroove	Expected	*χ*^2^ value	*p* value
M4-12 × M4-127	187	51	136	1:3	0.515	0.47
M4-18 × M4-120	183	40	143	1:3	0.96	0.32
M4-45 × M4-133	160	36	124	1:3	0.53	0.46

**Table 3:** Candidate genes in the fine mapping interval.

Gene ID	Physical location	Gene annotation
*MELO3C019699.2*	24,059,374. 24,062,605	Elongation factor 2
*MELO3C019698.2*	24,078,257. 24,093,312	Serine/threonine-protein kinase TNNI3K
*MELO3C019697.2*	24,109,474. 24,114,837	Elongation factor 2
*MELO3C019696.2*	24,119,712. 24,120,763	HVA22-like protein
*MELO3C035125.2*	24,127,294. 24,127,758	Transmembrane protein
*MELO3C035126.2*	24,129,470. 241,30,414	Myb/SANT-like DNA-binding domain protein
*MELO3C034863.2*	24,141,567. 24,141,728	Unknown protein
*MELO3C019694.2*	24,172,183. 24,177,539	AGAMOUS MADS box factor transcription factor
*MELO3C019691.2*	24,206,907. 24,211,844	Hexosyltransferase
*MELO3C019692.2*	24,211,247. 24,211,800	Unknown protein
*MELO3C035128.2*	24,234,163. 24,234,510	L10-interacting MYB domain-containing protein-like

Thirty-six pairs of primers that were uniformly distributed on the region identified by BSA-seq were designed. After polymorphism detection, 8 markers (6 CAPS and 2 InDel markers) were used for initial mapping. Fifty-one individuals with a recessive phenotype (grooved) were selected from the F_2_ generation in 2020 and genotyped with the 8 polymorphic markers. Ten out of the 51 plants that exhibited recombination events were used to reduce the BSA-seq region to approximately 1.71 Mb (from 2,300,980 to 24,713,146 bp) between the CAPS markers *Chr11_230070980* and *Chr11_24713146* and included 2 and 6 recombinants for each marker (Figure 2B).

To further narrow the initial mapping region, 1,200 F_2_ plants derived from M4-12 × M4-127 were planted in the spring of 2021. *Chr11_23007980* and *Chr11_24713146* were used as flanking markers to screen recombinant individuals (Figure 2B). Seventy-seven recombinant plants were obtained and transplanted to the greenhouse. Another 13 polymorphic KASP markers in the initial region were developed to genotype the recombinant plants. The genotype of the dominant recombinants was confirmed based on phenotypic segregation in their F_2:3_ families. Finally, the *Cmfsg* locus was delimited between the KASP markers *Chr11_24049463* and *Chr11_24256692* (approximately 207 kb of the physical distance, including 11 candidate genes) with two and two recombinants for each boundary marker, respectively (Figure 2C and Table 3).

### GWAS Identified the *Cmfsg* Gene on Chromosome 11

**Figure 3:**
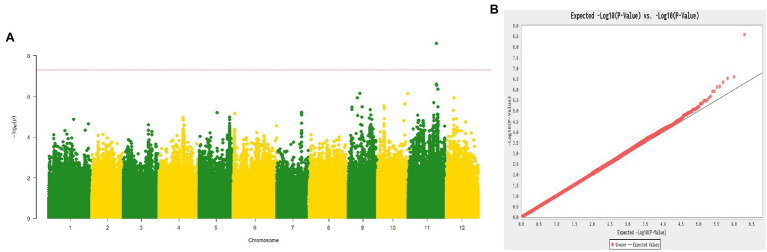
Manhattan plot **(A)** and Q-Q plot **(B)** of the melon fsg trait through a genome-wide association study.

According to the phenotype of plants grown in 2015 and 2016, the 187 melon lines consisted of 70 non-grooved lines and 117 grooved lines. The GWAS results revealed an obvious signal (approximately 1.23 Mb from 23,374,318 to 24,604,487 bp; reference genome: DHL 92 v3.5.1) associated with the fsg trait on chromosome 11 (Figures 3A,B). We also compared the 207-kb region to the genome reference DHL 92 v3.6.1, and this fine-mapping region was aligned from 24,049,540 to 24,256,769 bp, consistent with the biparental genetic mapping results.

### *In silico* BSA With Genome Resequencing Data From Other Melon Accessions Revealed That a Deleted 1.07-kb Chromosome Segment Correlated With Melon fsg

**Figure 4:**
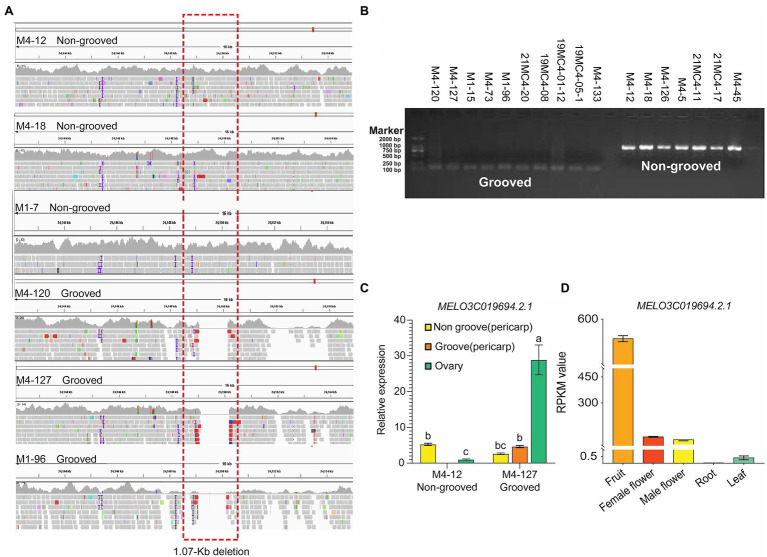
Candidate gene analysis. **(A)** Sequence alignment of the 1,070-bp deletion region from different materials at 23.85 kb upstream of *MELO3C019694.2.1*. **(B)** Bands of amplified *Chr11_24149068* from 11 melon materials. **(C)** The expression level of the gene in the parental ovary and the grooved and non-grooved pericarp of the mature fruit. **(D)** Gene transcription data from fruit, female flowers, male flowers, roots, and leaves.

The *Cmfsg* locus was finally mapped to a 207-kb region on chromosome 11 based on recombinant lines, but it was still a large interval. Although some recombinant plants were examined, no available markers were found in this segment according to the genome resequencing data from the parental lines. We also resequenced M4-120 and M4-18, and the results were the same as those obtained for M4-12 and M4-127; there were still no available markers in this region. Additionally, many gaps in this interval existed according to the reference genome data. The association analysis results also showed that the GWAS interval contained only four SNPs associated with the fsg trait. For the above reasons, we planned to use *in silico* BSA to compare the genome resequencing data from other melon accessions to detect variations related to fsg. By scanning the genome region among the M4-127 melon accessions, a 1.07 kb chromosome segment (chr11: 24,149,096-24,150,171) deletion was found in grooved melon but existed in nonfsg melon (Figure 4A). Beyond this variation, we did not find any other mutations in either the coding or promoter region in the 207-kb interval of the 11 candidate genes that could distinguish grooved and non-grooved melon accessions. We designed a PCR marker (Table 1) according to the resequencing data containing this 1.07-kb variation and amplified this fragment from 17 melon accessions. The results showed that all the grooved lines produced a 101-bp PCR product, while a 1,171-bp product was amplified from the non-grooved lines (Figure 4B). These results indicated that this 1.07-kb region is highly correlated with the fsg trait.

### Gene Expression Analysis Revealed CmMADS-Box as a Candidate Gene for the Melon fsg Trait

To predict the candidate gene for melon fsg, we focused on the chromosome region containing the 1.07-kb segment. The 1.07-kb deletion was upstream (approximately 23.8 kb) of *MELO3C019694.2* (annotated as AGAMOUS MADS-box transcription factor). We then detected the expression pattern of *MELO3C019694.2* in the ovary and the grooved and non-grooved pericarp tissues in the mature fruit between M4-127 and M4-12. The results showed that *MELO3C019694.2* expression in the ovary of the M4-127 grooved line was 28.8 times higher than that of the M4-12 non-grooved line, with a significant difference. In mature fruit from the M4-127 line, *MELO3C019694.2* expression in the grooved part (4.65 ± 0.32b) was 1.77 times higher than that in the non-grooved part (2.62 ± 0.22bc). There was no difference in the *MELO3C019694.2* expression level in the non-grooved part of the mature fruit of the two parents (Figure 4C). Combined with the transcriptome data from the Cucurbitaceae database (BioProject: PRJNA383830; Figure 4D), *MELO3C019694.2* was more specifically expressed in fruit than in female flowers, male flowers, roots, and leaves, suggesting that this gene may be involved in the regulation of melon fsg trait formation.

## Discussion

The fruit surface groove is an important quality of fruit appearance that contributes to its commercial value. The external morphology of fruits also plays an important role in plant evolution. The diversity of fruit shapes, including variations such as stripes, may promote seed transmission and plant evolution by attracting fruit eaters (Encinas-Viso et al., 2014). In this study, six parental lines were used to configure three genetic populations, and these populations were used to analyze the genetic inheritance of melon fsg. The results showed that the fsg was controlled by a major effective gene, this is consistent with the research results of Harel-Beja et al. (2010), Wang et al. (2018), and Zhao et al. (2019). Zhao et al. (2019) identified the same gene and variation region in the study of melon fruit suture. But the 1.07 kb was not co-segregated with fruit groove among the melon nature panel. A 122 (15.18%) of 807 non grooves had a deletion of 1.07 kb, and 42 (16.34%) of 257 grooves had no deletion of 1.07 kb indicated that 1.07 kb deletion maybe not the only reason for groove trait formation. Previous studies also showed that there were multiple SNPs loci (not only on chromosome 11, but also on chromosome 5 and 7 related with the depth of fruit groove) related to fsg traits (Hu et al., 2019). It indicates that there may be other genes controlling fsg traits in melon. Based on our GWAS panel, we found that the 1.07 kb region was not associated with subspecies and geographic distribution. The 1.07 kb region could also associate with this trait, whether the accessions belonged to the *C. agrestis* or *C. melo* with different geographical distribution (In Figures 4A,C, M1-7, M1-96, and M1-15 belonged to *C. agrestis*, others belonged to *C. melo*).

Coincidentally, Harel-Beja et al. (2010) found that the stripes in the young ovary and the surface grooves in the mature fruit located in the same position indicated that the two traits had a high correlation. Pumpkins, cucumbers, and gourds usually display stripes of different colors and shapes prominently (Goldman, 2004). The formation of stripes is the irregular accumulation of some pigments, such as carotenoids, flavonoids, and chlorophyll (Feder et al., 2015). Therefore, peel stripes are also known as the second color of peel (Cohen et al., 2014). Fruit surface groove also called vein tracks (Zhao et al., 2019). Generally, strong or dark stripes appear between the main veins on the fruit surface, and light stripes are located above or near the main veins (Paris, 2002). The position of this external stripe is consistent with the position of the main subepidermal vascular system of the ovary and fruit (Paris, 2020). It seemed that the position of stripes was just located in the suture/groove. But many melon accessions without groove still have the strips. So, we speculated that the stripes and fruit groove are two phenotypic characters.

*MELO3C019694.2* (annotated as AGAMOUS MADS-box transcription factor) was predicted as the most likely candidate gene for the melon fsg trait based on the expression patterns in the ovary, grooved, and non-grooved peel tissues in mature fruit from the parental lines in this study. The MADS-box family is an important transcription factor that plays an important role in plant growth and development and signal transduction (Zeng et al., 2018). In the process of evolution, the family experienced a series of gene duplication events, resulting in gene sub-functionalization or new functionalization and resulting in plant morphological diversity (Theissen et al., 2000). During plant growth and development, MADS-box genes may affect plant growth and development at different stages, parts, tissues, and organs (Hardenack et al., 1994). In addition to regulating flower development, the plant MADS box is also involved in the development of roots, leaves, fruits, seeds, and embryos (Garcia-maroto et al., 2003). Among the transcription factor networks that affect fruit ripening, MADS-box family transcription factors act as central regulators of ripening (Smaczniak et al., 2012; Gapper et al., 2013). In *Arabidopsis*, four genes, AG, SHATTERPROOF1 (SHP1), SHATTERPROOF2 (SHP2), and SEEDSTICK (STK), belong to the AG subfamily, of which SHP1 and SHP2 were previously called AGAMOUS-LIKE 1 and AGAMOUS-LIKE 5 (Li et al., 2016). Many studies have shown that the AGAMOUS (AG) gene, as a major regulator of floral organ differentiation and flowering decisions, can coordinate various cell fate decisions during flower development (Garceau et al., 2017). Studies on *Arabidopsis* found that the expression of SHP 1/2 is strictly localized in the marginal area of the valve by inhibiting FRUITFULL (FUL) expressed in the adjacent valve area and inhibiting REPLUMLESS (RPL) expressed in the adjacent valve area, which leads to silique cracking (Liljegren et al., 2000). FUL is responsible for the normal division and proliferation of cells in the valve, while SHP1 and SHP2 control the differentiation of the split zone (Liljegren et al., 2004). SHP promotes the expression of INDEHISCENT (IND) and ALCATRAZ (ALC; Rajani and Sundaresan, 2001). Tomato AGAMOUS-LIKE 1 (*TAGL1*) is a MADS-box transcription factor gene. The study found that *TAGL1* is homologous to the repeat genes SHP1 and SHP2 of *Arabidopsis thaliana* (Wei et al., 2020). RNA interference (RNAi) inhibition of TAGL1 in tomato fruit resulted in inhibited fruit ripening and reduced pericarp thickness, suggesting the existence of a molecular bridge linking pulp pericarp development and fruit ripening (Fujisawa et al., 2014). However, there are large differences in the copy number of MADS-box genes in different species, resulting in the sub-functionalization of many parallel homologous genes (Smyth, 2018). Therefore, the most significant feature of the MADS-box gene family is that it is involved in the process of fruit development in different plants. The roles vary widely among family members. A comparative analysis of *Brassica* plants shows that changes in the expression of the MADS-box transcription factor can control the change from cracking to noncracking in fruits of related species (Muhlhausen et al., 2013; Carey et al., 2019). This may also be the reason why melons with the characteristics of fruit surface grooves do not easily crack, but the specific regulatory mechanism needs further research.

To identified if there existed some conserved domain in the 1.07 kb region, we extracted the sequences of the 1.07 kb and making a BLAST alignment. Unfortunately, we did not find any conserved domain. There were still 23.8 kb located between the 1.07 kb segment and the *MELO3C019694.2*, but still contain many gaps according to the reference genome. So there may exist some other gene in this 23.8 kb region regulate the groove trait formation. However, in addition to this mutation, we did not find any other mutations in the coding region or promoter region of 207 kb of 11 candidate genes. Much more information was needed in the further research, such as a comprehensive reference genome data, a more precise genetic mapping to confirm the candidate gene. Therefore, the mechanism of fsg trait formation will be the focus of future research.

## Data Availability Statement

The original contributions presented in the study are included in the article/**Supplementary Material**, further inquiries can be directed to the corresponding authors.

## Author Contributions

XD performed the major experiments. HL and ZZ serviced generations production. ShuL, ZS, LX, and JZ offered germplasm resources. ShiL designed the experiment and analyzed the data. FL and ShiL revised the manuscript and are the co-corresponding authors. All authors contributed to the article and approved the submitted version.

## Funding

This work was supported by funding from the National Natural Science Foundation of China (grant no. 31972436 and 32030094) by the China Agriculture Research System of MOF and MARA (grant no. CARS-25), and by Taishan Industrial Leading Talents Project (grant no. LJNY202112).

## Conflict of Interest

JZ is employed by Qinggang Ruixue Agriculture Co., Ltd. ShuL, ZS, and LX are employed by Shouguang Sanmu Seeding Co., Ltd.

The remaining authors declare that the research was conducted in the absence of any commercial or financial relationships that could be construed as a potential conflict of interest.

## Publisher’s Note

All claims expressed in this article are solely those of the authors and do not necessarily represent those of their affiliated organizations, or those of the publisher, the editors and the reviewers. Any product that may be evaluated in this article, or claim that may be made by its manufacturer, is not guaranteed or endorsed by the publisher.

## Supplementary Material

The Supplementary Material for this article can be found online at: https://www.frontiersin.org/articles/10.3389/fpls.2022.828287/full#supplementary-material

Supplementary Figure S1 Transverse sections of the groove and non-groove F_2_ individuals.

## Footnotes

^1^ https://tassel.bitbucket.io/

^2^ http://cucurbitgenomics.org/ftp/genome/melon/v3.5.1/

^3^ https://cran.r-project.org/web/packages/qqman/index.html

^4^ http://www.igv.org/
