# Supplementary figures and images for: Identification of Candidate Chromosome Region Related to Melon (Cucumis melo L.) Fruit Surface Groove Trait Through Biparental Genetic Mapping and Genome-Wide Association Study
Source: Front Plant Sci. 2022 Apr 5;13:828287. doi: 10.3389/fpls.2022.828287 (PMC9022103; doi:10.3389/fpls.2022.828287)

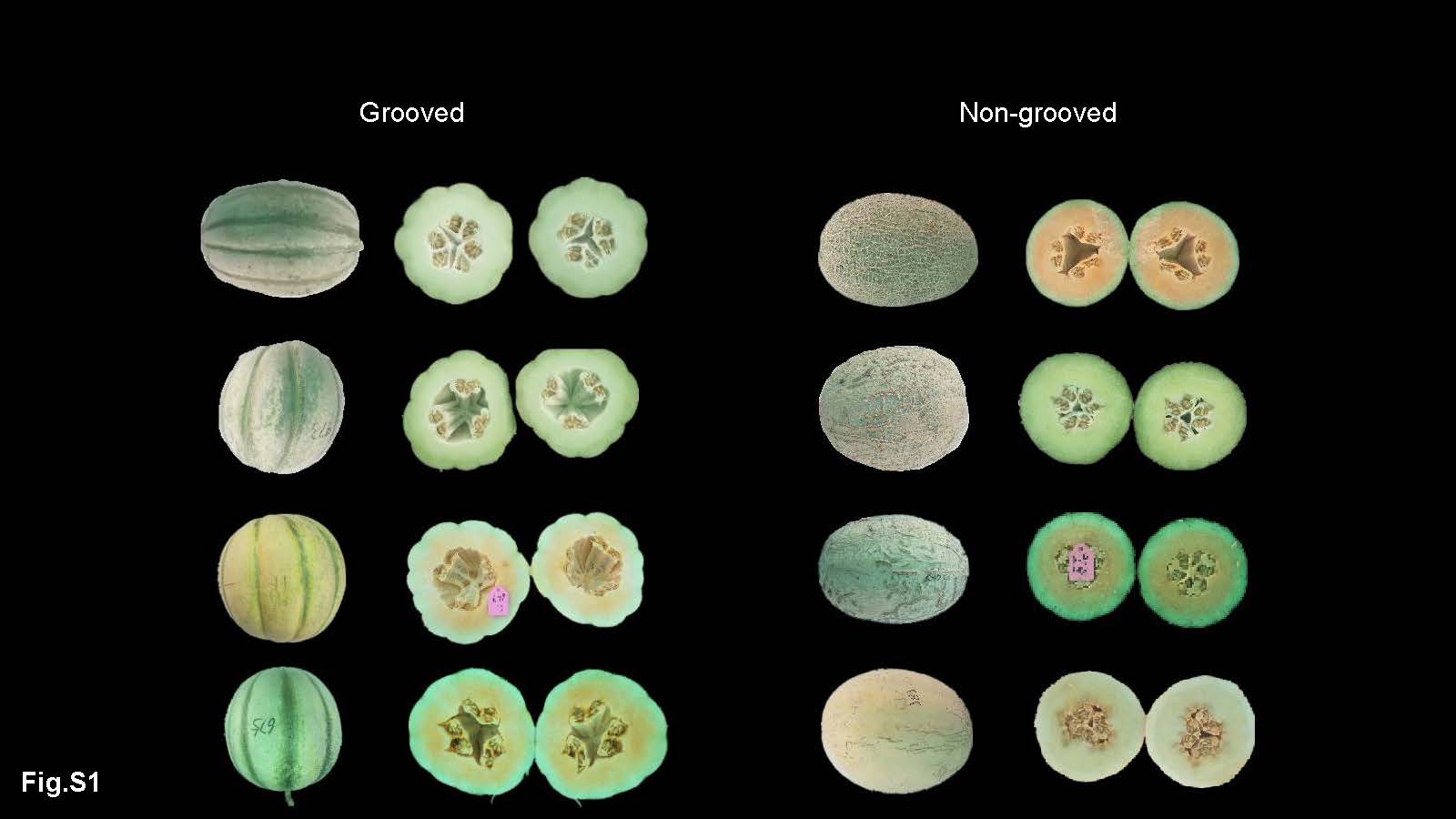

Supplement: Supplementary Figure S1 — Transverse sections of the groove and non-groove F2 individuals. [file Image_1.JPEG]
